# Taste preference changes throughout different life stages in male rats

**DOI:** 10.1371/journal.pone.0181650

**Published:** 2017-07-25

**Authors:** Chizuko Inui-Yamamoto, Takashi Yamamoto, Katsura Ueda, Michiko Nakatsuka, Shunji Kumabe, Tadashi Inui, Yasutomo Iwai

**Affiliations:** 1 Department of Oral Anatomy, Osaka Dental University, Hirakata, Osaka, Japan; 2 Department of Health and Nutrition, Faculty of Health Science, Kio University, Kitakatsuragi-gun, Nara, Japan; 3 Division of Behavioral Physiology, Department of Behavioral Sciences, Graduate School of Human Sciences, Osaka University, Suita, Osaka, Japan; The University of Tokyo, JAPAN

## Abstract

Taste preference, a key component of food choice, changes with aging. However, it remains unclear how this occurs. To determine differences in taste preference between rats in different life stages, we examined the consumption of taste solutions and water using a two-bottle test. Male Sprague-Dawley rats of different ages were used: juvenile (3–6 weeks), young adult (8–11 weeks), adult (17–20 weeks), middle-aged (34–37 weeks), and old-aged (69–72 weeks). The intakes of the high and low concentration solutions presented simultaneously were measured. We observed that the old-aged group had lower preference ratios for 0.3 M sucrose and 0.1 M MSG in comparison with other groups. The preference ratio for 0.03 mM QHCl was higher in the middle-aged group than in the three younger groups and higher in the old-aged group than the juvenile group. The taste preferences for HCl and NaCl did not significantly differ among the age groups. The old-aged group tended to prefer high concentrations of sucrose, QHCl, NaCl, and MSG to low concentrations, indicating age-related decline in taste sensitivity. We also aimed to investigate differences between life stages in the electrophysiological responses of the chorda tympani nerve, one of the peripheral gustatory nerves, to taste stimuli. The electrophysiological recordings showed that aging did not alter the function of the chorda tympani nerve. This study showed that aging induced alterations in taste preference. It is likely that these alterations are a result of functional changes in other peripheral taste nerves, the gastrointestinal system, or the central nervous system.

## Introduction

Humans and animals normally prefer sweet, salty, and umami tastes to sour and bitter tastes. However, taste preferences are easily changed by postnatal factors, including learning, environment, and nutritional status. Furthermore, aging itself may result in alterations in taste preference, as aging is generally accompanied by certain changes in bodily tissues and functions. Evidence suggests that taste sensitivity to sucrose is lower in older people [1–7]. Dietary and energy requirements change throughout the various life stages [8–10], and basal metabolic rate decreases with age in a near-linear manner [11, 12]. The observed decrease in taste sensitivity with aging could result in older people eating more foods with stronger flavor and, possibly, higher calories; this could contribute to the development of lifestyle-related diseases. A better understanding of age-related changes in taste preference may be important for disease prevention.

Aged (90 weeks old) Fischer-344 rats showed significantly lower intake of food and sucrose than those aged 20–35 weeks [13]. Decreases in taste sensitivity were observed in male Sprague-Dawley rats aged 28 months (112 weeks) [14]. These studies suggest the possibility of altered taste thresholds or taste preferences with aging in rodents. The animals in these studies were given taste stimuli in both younger and older age periods as part of the within-subject experimental design. Therefore, it is possible that not only alterations in physiological function but also in consumption experience caused the differences in taste preferences across age groups. On the other hand, two reports investigated the differences in taste preference between two separate groups (Sprague-Dawley rats aged 5–12 weeks and 21–22 months; B6C3F1/J mice aged 10 and 18 weeks), in which the older animals were naïve to the taste stimuli until reaching the experimental age [15, 16]. These studies revealed a significant reduction of umami preference in the older rats [15], and of sucrose preference in the older mice [16]. Thus, it is likely that taste preferences decrease with aging independent of consumption experience.

Humans and animals have several major stages in their lifetimes, including weaning, reproduction, and old age. Transitions between stages are accompanied by changes in dietary and energy requirements, as well as alterations in hormone secretion. These provide the possibility that age-related changes in taste preferences are stepwise rather than abrupt. In order to elucidate whether aging induces graded shifts in taste preference, we assessed differences in the consumption of taste solutions (sucrose, saccharin, NaCl, HCl, quinine HCl, and monosodium glutamate) among five age-separated groups (juvenile, young adult, adult, middle-aged, and old-aged), which were naïve to the taste stimuli before reaching the experimental age.

Although many studies have examined the alteration of taste preferences with aging in humans and animals, its underlying mechanisms remain unclear. Gustatory information is transmitted from the tongue to the central nervous system via the taste nerves, including the chorda tympani nerve, the glossopharyngeal nerve, the superior laryngeal nerve, and the greater superficial petrosal nerves in the oral cavity. Previous studies have shown that taste experience during development influences the function of the chorda tympani nerve [17–20]. These developmental changes in the function of the peripheral gustatory system suggest that aging results in altered taste nerve activity during different life stages. Therefore, using electrophysiology, we examined the effect of aging on the responses of the chorda tympani nerve (which transmits gustatory information from the anterior tongue to the brainstem) to taste stimuli.

## Materials and methods

### Age-related changes in taste preference using a 48-h two-bottle test

A 48-h two-bottle test, a standard behavioral test for taste preference, was conducted on 46 male Sprague-Dawley rats (CLEA Japan, Inc., Japan) between 3 and 72 weeks of age and weighing 95–1050 g. Rats generally have a mean lifespan of 2–3 years that includes two critical time-points: the end of weaning and reproduction. Therefore, we divided the rats into five groups: juvenile (3–6 weeks, just after weaning, n = 9), young adult (8–11 weeks, early reproductive phase, n = 8), adult (17–20 weeks, late reproductive phase, n = 9), middle-aged (34–37 weeks, end of reproduction, n = 10), and old-aged (69–72 weeks, n = 10). We did not use rats over 74 weeks of age because they carry a high risk of spontaneous disease. The experience of consuming taste solution is likely to have an effect on subsequent ingestive behaviors. To avoid this, a different set of rats was used for each age group. The juvenile group was purchased at 3 weeks. The young adult and the adult groups were purchased one week before the behavioral experiments. As rats of more than 30 weeks old were not available for purchase, the middle-aged and the old-aged groups were purchased at 30 weeks and raised until the appropriate age for testing in the animal-breeding facilities of the faculty. All rats were allowed food pellets (MF, Oriental Yeast, Osaka, Japan) and distilled water (DW) ad libitum, and handled by the experimenters every day before attaining the appropriate age. Animals were housed individually in plastic cages suitable for their body mass: 225 × 338 × 140 mm for rats 3–11 weeks old, and 345 × 403 × 177 mm for rats 17–72 weeks old. Cages were changed once a week. Since environmental changes could alter animals’ consumption behavior, the taste stimulus was presented after at least 60 hours of acclimation in the new plastic cage.

The ambient temperature was maintained at 23°C in a 12:12 h light/dark cycle (lights on between 8:00 and 20:00). All animal care and experimental guidelines conformed to those established by the National Institutes of Health and were approved by “Guide for the Care and Use of Laboratory Animals” in the Osaka Dental University Animal Care and Use Committee (Permit Number: 12–02045).

After the acclimation, all rats were presented with two bottles in their home cages: one containing DW and the other containing a taste solution. The bottle consisted of a 100-ml plastic syringe (JS-S00S, JMS Co., Ltd, Tokyo, Japan) and a stainless steel spout (TV-25, CLEA, Tokyo, Japan). The rats could freely access both bottles and chow for 48 h. To avoid positional preference, the positions of the bottles were switched 24 h after the start of the presentation. We recorded 48-h fluid consumption by measuring the weight of the bottles. The taste solutions were sucrose (0.3 and 0.5 M), sodium saccharin (saccharin, 5 mM), NaCl (0.1 and 0.3 M), QHCl (0.03 and 0.3 mM), MSG (0.1 M), and HCl (10 and 50 mM). To exclude the possibility of order effects, the taste solutions were presented in pseudorandom order, without grouping similar solutions by concentration. In addition, the presentation order was different among different rats. The order was one of the following: 1) 0.3 M sucrose, 0.1 M NaCl, 0.3 mM QHCl, 5 mM saccharin, 50 mM HCl, 0.1 M MSG, 0.03 mM QHCl, 0.5 M sucrose, 0.3 M NaCl and 10 mM HCl; 2) reverse order of 1); 3) 0.1 M MSG, 50 mM HCl, 5 mM saccharin, 0.3 mM QHCl, 0.1 M NaCl, 0.3 M sucrose, 0.03 mM QHCl, 0.5 M sucrose, 0.3 M NaCl and 10 mM HCl. We spent 4 weeks (e.g., 3–6 weeks of age in the juvenile group) completing the presentation of all 10 taste solutions.

### Age-related changes in preference for low and high concentrations of taste solutions using a 48-h two-bottle test

It was possible that the differences in the consumption of the taste solutions and water across the life stages were due to the changes in the taste thresholds. In order to answer to this question, we investigated the intake of low and high concentrations of taste solutions in the second experiment. It included a new series of 38 male Sprague-Dawley rats (CLEA Japan, Inc., Japan) aged 3–72 weeks and weighing 125–980 g. We divided the rats into five groups as in the first experiment: juvenile (n = 8), young adult (n = 8), adult (n = 7), middle-aged (n = 7), and old-aged (n = 8). The housing conditions were the same as described above.

All rats were presented with two bottles containing the same taste solution for 48 h, one at a high concentration and the other a low concentration. The taste solutions were 0.3 M vs. 0.5 M sucrose, 5 mM vs. 50 mM saccharin, 0.03 mM vs. 0.3 mM QHCl, 0.1 M vs. 0.3 M NaCl, 0.1 M vs. 0.3 M MSG, and 10 mM vs. 50 mM HCl. The taste solutions were presented in pseudorandom order. The order was one of the following: 1) 0.3 and 0.5 M sucrose, 0.1 and 0.3 M NaCl, 5 mM and 50 mM saccharin, 0.1 M and 0.3 M MSG, 0.03 mM and 0.3 mM QHCl, and 10 mM and 50 mM HCl; 2) reverse order of 1); 3) 5 mM and 50 mM saccharin, 0.1 and 0.3 M NaCl, 0.3 and 0.5 M sucrose, 0.1 M and 0.3 M MSG, 0.03 mM and 0.3 mM QHCl, and 10 mM and 50 mM HCl. Rats from this test were subsequently used in the electrophysiological experiments.

### Electrophysiological measurements of the responses of the chorda tympani nerve to taste solutions

The rats were anesthetized with an intraperitoneal injection of 60 mg/kg sodium pentobarbital (Somnopentyl^®^; Kyoritsu Seiyaku, Tokyo, Japan). Supplementary injections of 0.3 g/kg urethane were administered as needed to maintain a surgical level of anesthesia. A tracheal cannula was implanted and the animal properly secured within a head holder. The chorda tympani nerve was cut near its entrance into the tympanic bulla and dissected free from the underlying tissues. An indifferent electrode was positioned nearby in the wound. The whole-nerve activity was amplified, displayed on an oscilloscope, and monitored using an audio amplifier. The amplified signals were passed through an integrator with a time constant of 0.3 s and displayed on a slip chart recorder.

After confirmation of stable recording, we applied 5 ml of taste solution to the rat’s tongue for 30 s. The rat’s tongue was rinsed with DW after completion of taste stimulation. We measured the entire integrated response during the simulation as the whole nerve response. In electrophysiological experiments it is possible that any endogenous or exogenous factors may produce individual differences in the recording of neural activities. Therefore, we normalized the taste responses by dividing the magnitudes of the responses to each taste stimuli by the response to 0.1 M NH_4_Cl, which is generally used as a standard stimulus in electrophysiological recordings of peripheral taste nerves.

### Statistical analysis

Normalized food intake was calculated by dividing the 24-h food intake by BW (per 100 g) and analyzed using one-way analysis of variance (ANOVA) and post-hoc Tukey HSD tests. The data from the electrophysiological experiments were also analyzed using one-way ANOVA.

The previous studies investigating drinking behavior generally used 24-h intake as the behavioral index. To enable a comparison of our results with the prior studies, we calculated the 24-h intake volume as half of the 48-h intake volume. The taste solution preference ratios in the first experiment were calculated by dividing the volume of taste solution ingested by the total intake of DW + taste solution, and analyzed using one-way ANOVA with Tukey's HSD post-hoc tests. The preference ratios for higher concentration taste solutions were calculated by dividing the intake of the higher concentration solution by the total intake of higher + lower concentration solutions, and analyzed using one-way ANOVA with Duncan’s post-hoc tests. We also analyzed whether the preference ratios were significantly different from chance level (0.5) using an independent t-test. The difference between net intake of DW and each taste solution for each age group was analyzed by paired t-test. All statistical analyses were performed using Statistica software (StatSoft, Inc., Tulsa, OK, USA). A *P* value < 0.05 was considered significant.

## Results

### Age-related changes in taste preference using a 48-h two-bottle test

**Fig 1:**
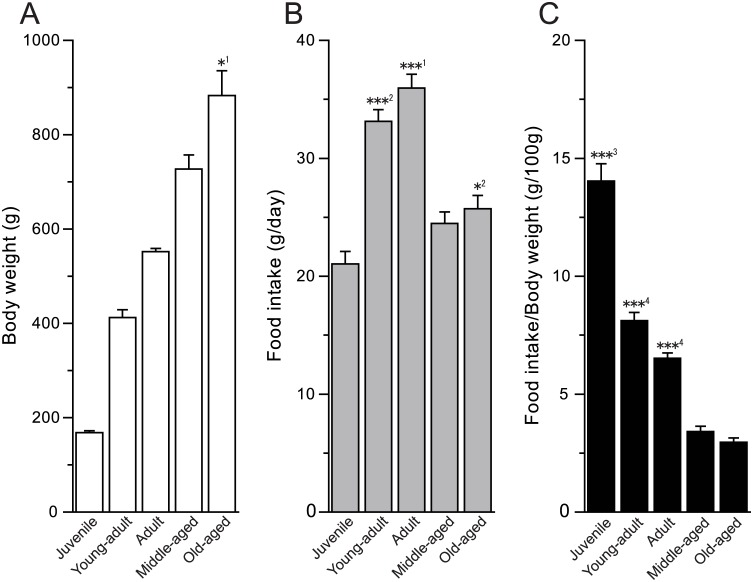
General index. (A) Body weight was greater in the old-aged group compared to other groups (*^1^*P* < 0.05); (B) The young adult and adult groups had greater 24-h food intake than other groups (***^1^ *P* < 0.001, adult vs. juvenile, middle-aged, and old-aged; ***^2^ *P* < 0.001, young adult vs. juvenile, middle-aged, and old-aged; *^2^*P* < 0.05, juvenile vs. old-aged); (C) Food intake based on body weight (per 100 g) was greater in the juvenile group than in other groups (***^3^ *P* < 0.001). Food intakes based on body weight of the young adult or adult group was greater than those of the middle-aged and old-aged groups (***^4^ P <0.001). Data is presented as mean + SEM.

Rats gradually grow larger from post-weaning to before the end of reproduction. Because body size is closely related to nutritional requirements, we assessed differences in BW and food and fluid consumption among the different age groups (Fig 1). Rats in the old-aged group weighed significantly more than rats in other groups (*P* < 0.05). The net food intake of the old-aged group was less than that of the young adult and adult groups (*P* < 0.001 for both) but was significantly greater than that of the juvenile group (*P* < 0.05). However, the normalized food intake values revealed lower food intake relative to BW in the old-aged rats than in the juvenile rats (*P* < 0.001).

**Fig 2:**
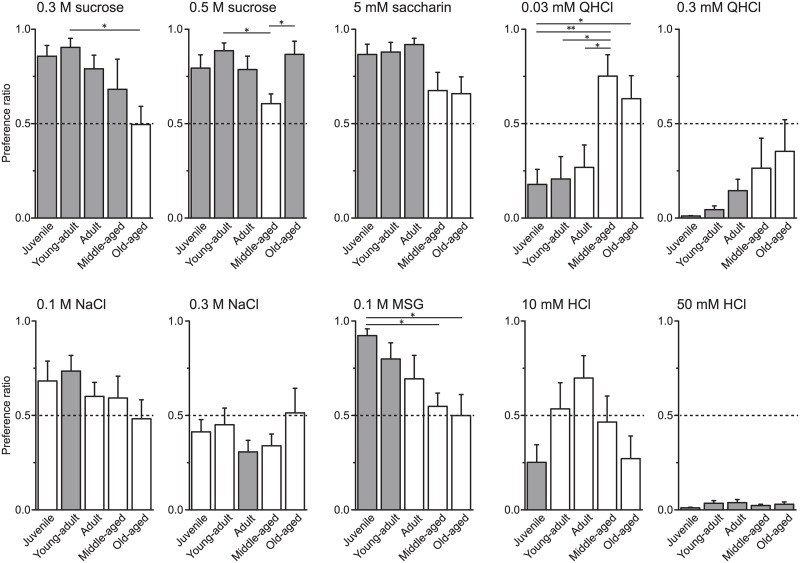
Age-related changes in taste preference using a 48-h two-bottle test. Mean preference ratios + SEM for each taste solution. The preference ratios for 0.3 M sucrose, 0.5 M sucrose, 0.03 mM QHCl, and 0.1 M MSG significantly differed among groups. *P < 0.05, young adult vs. old-aged (0.3 M sucrose), middle-aged vs. young adult and old-aged (0.5 M sucrose), juvenile vs. old-aged, middle-aged vs. young adult and adult (0.03 mM QHCl), juvenile vs. middle-aged and old-aged (0.1 M MSG); **P < 0.01, juvenile vs. middle-aged (0.03 mM QHCl). The dotted lines indicate chance level. The gray-colored bars indicate preference ratios significantly different from the chance level. The *P* value of the middle-aged group in the 0.03 mM QHCl experiment was 0.06.

Fig 2 shows how the preference ratio for taste stimuli differed among age groups. The juvenile and young adult groups exhibited similar preferences for several taste stimuli, each drinking much more sweet and umami solutions than DW and avoiding bitter and strongly sour tastes. In contrast, the middle-aged group demonstrated different preferences, with lower preference ratios for sweet and umami tastes and higher preference ratios for bitter tastes compared to the younger three groups. The old-aged group showed a lower preference ratio for 0.3 M sucrose and 0.1 M MSG (but not 0.5 M sucrose), and higher preference ratio for 0.03 mM QHCl compared to the juvenile group. One-way ANOVA revealed significant main effects of age with regard to the following taste solutions: 0.3 M sucrose (F(4, 35) = 2.91, *P* < 0.05), 0.5 M sucrose (F(4, 35) = 3.19, *P* < 0.05), saccharin (F(4, 35) = 3.16, *P* < 0.02), 0.03 mM QHCl (F(4, 35) = 5.64, *P* < 0.01), and MSG (F(4, 35) = 3.70, *P* < 0.05). Post-hoc analyses demonstrated a significantly lower preference ratio for 0.3 M sucrose in the old-aged group than the young adult group (*P* < 0.05). Preferences for 0.5 M sucrose in the middle-aged group were significantly lower than that in the young adult and the old-aged groups (*P* < 0.05). The older two groups (middle-aged and old-aged) exhibited significantly lower preference ratios for 0.1 M MSG than the juvenile group (*P* < 0.05 for both). The middle-aged group had significantly greater preference ratios for 0.03 mM QHCl compared to the three younger groups (middle-aged vs. juvenile, *P* < 0.01; vs. young adult and adult, *P* < 0.05). The old-aged group also had it compared to the juvenile group (old-aged vs. juvenile, *P* < 0.05).

**Fig 3:**
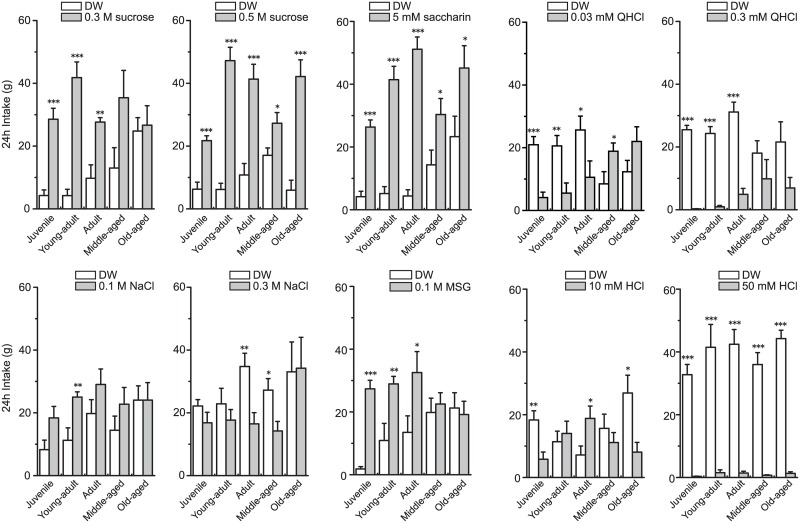
The 24-h intake for DW and each solution. Mean 24-h intake + SEM from the two-bottle preference test in which two solutions were presented simultaneously for 48 h. All asterisks indicate comparisons between the taste substance and DW. *P < 0.05, **P < 0.01, ***P < 0.01.

The preference ratios indicate which solution the animals preferred but do not indicate the net intake of solutions. As shown in Fig 1, the rats exhibited age-dependent decreases in food intake based on BW. As solution intake volumes typically correlate with pellet intake, it seemed likely that the different pellet consumption behaviors between age groups influenced the age-related differences in preference ratios. Therefore, in Fig 3, we show the net intake of DW and taste solutions. The juvenile, young adult, and adult groups consumed significantly more of the sucrose, saccharin, and MSG solutions (juvenile, *P* < 0.001 for all solutions; young adult, *P* < 0.001 for sucrose and saccharin, *P* < 0.01 for MSG; adult, *P* < 0.001 for 0.5 M sucrose and saccharin, *P* < 0.01 for 0.3 M sucrose, *P* < 0.05 for MSG). The young adult group also consumed more 0.1 M NaCl than DW (*P* < 0.01). The adult group consumed significantly less 0.3 M NaCl than DW. On the other hand, the younger three age groups (juvenile, young adult, and adult) consumed less QHCl and 50 mM HCl than DW (juvenile, *P* < 0.001 for all solutions; young adult, *P* < 0.001 for 0.3 mM QHCl and 50 mM HCl, *P* < 0.01 for 0.03 mM QHCl; adult, *P* < 0.001 for 0.3 mM QHCl and 50 mM HCl, *P* < 0.05 for 0.03 mM QHCl). The juvenile group also consumed significantly less 10 mM HCl than DW (*P* < 0.01). In contrast to the younger three groups, the middle-aged and old-aged groups did not consume significantly more 0.3 M sucrose and MSG or less 0.3 mM QHCl than DW.

### Age-related changes in taste preference for low or high concentrations of taste solutions

In this behavioral experiment, the rats were simultaneously presented with taste solutions at low and high concentrations. Even when a taste is appetitive (e.g. sweet, salty, or umami), a taste stimulus that is too strong will not be pleasant. It was hypothesized that older animals would exhibit a greater preference for higher concentrations if aging elevated the taste threshold.

**Fig 4:**
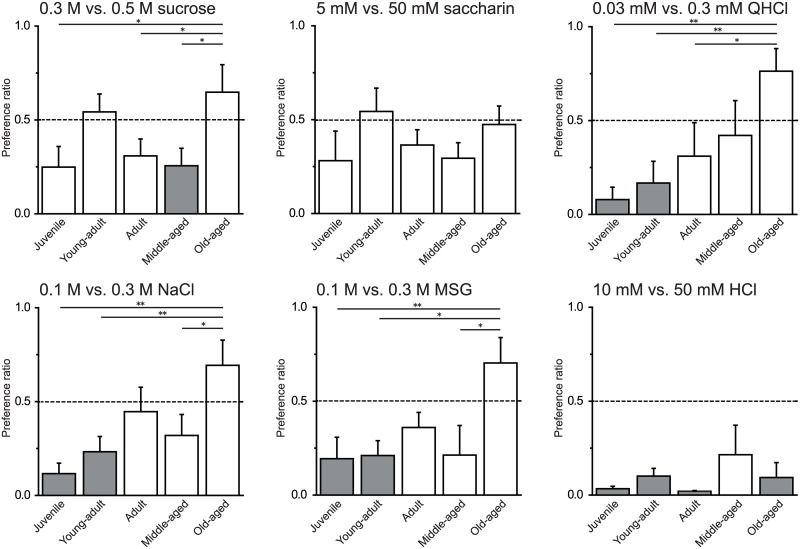
Age-related changes in taste preference for low or high concentrations of taste solutions. Mean preference ratios + SEM for taste solutions of higher concentrations. The old-aged group exhibited a significantly greater preference for the higher concentration solution of sucrose (*P < 0.05, vs. juvenile, adult, and middle-aged), QHCl (**P < 0.01, **P < 0.01, and *P < 0.05 vs. juvenile, adult, and young-adult, respectively), NaCl (**P < 0.01, **P < 0.01, and *P < 0.05 vs. juvenile, young-adult, and middle-aged, respectively), and MSG (** *P* < 0.01, *P < 0.05, and *P < 0.05 vs. juvenile, young-adult, and middle-aged, respectively). The dotted lines indicate chance level. The gray-colored bars indicate preference ratios significantly different from the chance level. The *P* value of the old-aged group in the 0.03 mM vs. 0.3 mM QHCl experiment was 0.08.

Our results demonstrated age-related differences in the preference for higher concentration taste solutions (Fig 4). Only the oldest age group exhibited a much greater preference for the higher concentration solutions than the lower concentration solutions. One-way ANOVA showed a main effect of group for the following taste solutions: sucrose (F (4, 29) = 2.86, *P* < 0.05), QHCl (F (4, 26) = 4.12, *P* < 0.05), NaCl (F (4, 27) = 4.38, *P* < 0.01), and MSG (F (4, 27) = 3.25, *P* < 0.05). Post-hoc analysis revealed that the old-aged group significantly preferred the higher concentration solutions of sucrose (vs. juvenile, adult, and middle-aged: *P* < 0.05), QHCl (vs. juvenile, adult, and young adult: *P* < 0.01, *P* < 0.01, and *P* < 0.05, respectively), NaCl (vs. juvenile, young adult, and middle-aged: *P* < 0.01, *P* < 0.01, and *P* < 0.05, respectively), and MSG (vs. juvenile, young adult, and middle-aged: *P* < 0.01, *P* < 0.05, and *P* < 0.05, respectively). The preference ratio of the old-aged group did not differ from the young adult group for sucrose, or from the adult group for NaCl and MSG. However, the preference ratios of the young adult and adult groups were approximately 0.5 (chance level). These findings indicate that only the old-aged group preferred the high concentrations of sucrose, QHCl, NaCl, and MSG to the low concentration solutions.

### Electrophysiological experiments with the chorda tympani nerve

**Fig 5:**
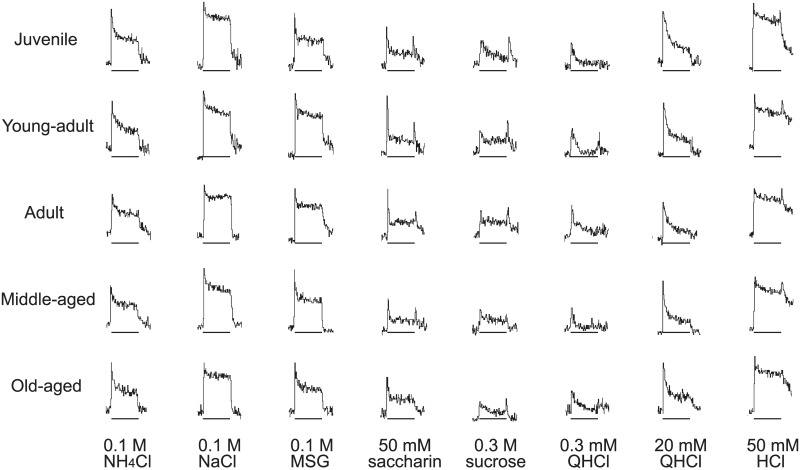
A representative integrated response of the chorda tympani nerve to 0.1 M NH_4_Cl, 0.1 M NaCl, 0.1 M MSG, 50 mM saccharin, 0.3 M sucrose, 0.3 mM QHCl, 20 mM QHCl, and 50 mM HCl for each age group. The horizontal bar indicates a stimulus duration of 30 s.

**Fig 6:**
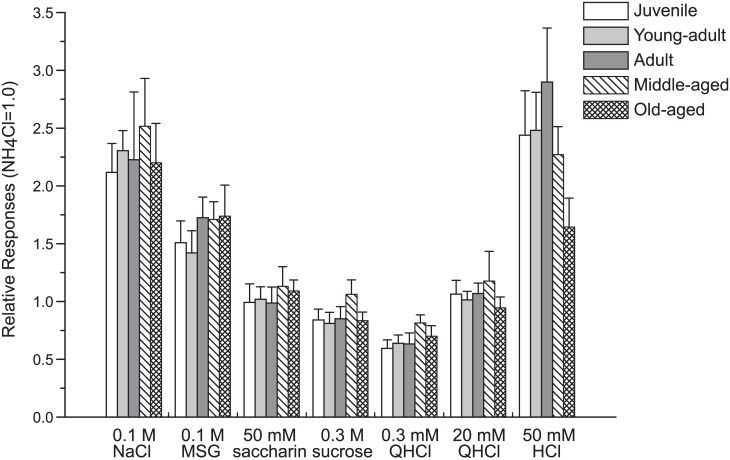
The relative responses to 0.1 M NH_4_Cl of the chorda tympani nerve to taste solutions. Mean relative responses of the chorda tympani nerve (+ SEM) to 0.1 M NaCl, 0.1 M MSG, 50 mM saccharin, 0.3 M sucrose, 0.3 mM QHCl, 20 mM QHCl, and 50 mM HCl. The responses to each taste solution did not significantly differ among age groups.

Based on previous studies, we used 0.1 M NaCl, 0.1 M MSG, 50 mM saccharin, 0.3 M sucrose, 0.3 mM QHCl, 20 mM QHCl, and 50 mM HCl in the electrophysiological experiments. We used the higher concentration of QHCl because the responses of the chorda tympani nerve to the lower concentration of QHCl have been reported to be very small [21]. Fig 5 presents examples of the chorda tympani nerve gustatory responses, clearly showing how the waveforms differed among the taste stimuli. However, significant group differences are difficult to observe. To compare the magnitude of the responses, we show the normalized response magnitude in Fig 6. One-way ANOVA revealed no main effects of group for any of the taste stimuli. These results suggest that aging does not affect gustatory processing in the peripheral nervous system.

## Discussion

In the present study, we examined the taste preferences and gustatory responses of the chorda tympani nerve in male Sprague-Dawley rats at different life stages. The behavioral experiments revealed that the old-aged group showed significantly lower preference for 0.3 M sucrose than the young adult group, and for 0.1 M MSG than the juvenile group. In contrast, the old-aged group demonstrated significantly higher preference for 0.03 mM QHCl than the juvenile group. The preference ratio for 0.1 M MSG in the middle-aged group was significantly lower than that in the juvenile group. The middle-aged group also displayed significantly higher preference for 0.03 mM QHCl than younger groups (juvenile, young adult and adult groups). When simultaneously presented with different concentrations of the same taste solution, only the old-aged rats drank larger volumes of the higher concentrations of sucrose, QHCl, NaCl, and MSG than the lower concentration solutions. However, the electrophysiological experiments revealed no significant differences between the different age groups with regard to the responses of the chorda tympani nerve, which is one of the peripheral taste nerves.

With simultaneous presentation of taste solution and DW, the old-aged group had a preference ratio of approximately 0.5 for 0.3 M sucrose, indicating that these rats drank similar volumes of 0.3 M sucrose and DW (Fig 3). The old-aged group also tended to have a lower preference ratio for saccharin. On the other hand, the old-aged group drank a much greater volume of 0.5 M sucrose than DW. These results suggest that the old-aged group had difficulty discriminating between DW and low-concentration solutions of sucrose or saccharin, indicating an age-related decline in sensitivities for the sweet taste.

As the old-aged group had a decreased preference for normally palatable (sweet and umami) taste and greater preference for aversive (bitter) taste compared to the other groups, the results indicate the possibility that the older rats could not detect the taste substances in the fluids. As shown in Figs 1 and 3, there were clear differences in food and water consumption among the groups, indicating that aging affects water and energy requirements. The lower food intake in the middle-aged (34–37 weeks) and old-aged (69–72 weeks) rats suggests an alteration in ingestive behavior by the end of reproduction. Therefore, we adopted another technique in which the rats were simultaneously presented with high and low concentrations of taste solutions. If aging causes deficits in taste detection, the old-aged rats may not be able to discriminate between high and low concentrations. The comparison between low and high concentrations of the same taste solutions revealed that the juvenile, adult, and middle-aged groups drank less 0.5 M sucrose than 0.3 M sucrose. Though sucrose is normally a palatable taste stimulus, animals exhibit a decreased preference for sucrose at high concentrations [22]. However, in the present study the old-aged rats showed a higher preference ratio to 0.5 M than 0.3M sucrose. These results further support the reduced sweet taste sensitivity in the older rats compared to the younger rats.

As shown in Fig 2, the old-aged group had a lower preference ratio for 0.1 M MSG than the younger groups. Furthermore, only the old-aged group preferred the high (0.3 M) concentration of MSG to the low (0.1 M) concentration. Miura et al. [15] reported reduced umami preference in aged Sprague-Dawley rats (21–22 months) compared to a young adult group (5–12 weeks). The umami receptor is a heterodimer of taste receptor type 1 member 1 and 3 (T1R1/T1R3), whereas the sweet receptor comprises taste receptor type 1 member 2 and 3 (T1R2/T1R3) [23]. Although there are similarities in the peripheral transduction, Sprague-Dawley rats can reportedly discriminate between umami taste and some sweet tastes [24]. These results suggest that the old-aged group also had a reduced sensitivity for MSG compared to the other age groups. In addition, it is suggested that amino acid receptors exist in the duodenum and intestine. Niijima [25] showed the increased discharge rate of the gastric branch of the vagus nerve by intestinal stimulation with isotonic MSG, but not NaCl, solution. These results suggest that a long-term presentation of MSG likely produce postingestive effects. Since it seems that aging cause decreased functions of the duodenum and intestine, the low MSG preference in the old aged group might be due to aging-induced changes in postingestive effects. We also found that the old aged rats preferred higher concentrations of NaCl and MSG than lower ones. If only the declined postingestive effects lower taste preferences of NaCl and MSG, the old aged rats should avoid the intake of the higher concentration of NaCl and MSG. Therefore, it is likely that the aging-induced changes in not only the postingestive effects but also the functions of the gustatory nerves other than chorda timpani and central nervous system result in the alteration in the taste preferences of NaCl and MSG.

Animals and humans normally dislike bitter tastes. However, the middle-aged and old-aged groups in our study had higher preference ratios (> 0.5) for the 0.03 mM QHCl solution compared to the other age groups (Fig 2), indicating that the two oldest groups drank substantially more QHCl than DW. The total intake volumes of 0.03 mM QHCl and DW in the older groups did not significantly differ from the intake volumes in the younger three groups (Fig 3), suggesting that the higher preference ratios for the low concentration QHCl solution in the older groups were not due to abnormal fluid consumption. When the older groups were presented with the high concentration QHCl solution, they had preference ratios of < 0.5. Our findings suggest that the older rats had a low preference for bitter tastes, as well as sweet and umami tastes. Even with a reduced preference for bitter tastes, the older rats were still expected to drink less high concentration QHCl solution, which is normally an aversive taste stimulus [26]. Surprisingly, the preference ratio for the higher to lower concentration solution of QHCl in the old-aged group was > 0.5 (Fig 4). This indicates that rats in the old-aged group preferred 0.3 mM to 0.03 mM QHCl solution. On the other hand, although the middle-aged rats preferred 0.03 mM QHCl to DW (Fig 2), they did not show preference for higher concentration QHCl. These results suggest that the rats of 34 weeks or older (middle- and old-aged rats) have less ability to detect bitterness or feel unpleasantness for quinine. Moreover, it is assumed that the old-aged rats are unable to detect the differences in the concentration of the taste solutions.

We expected our results to show age-related changes in the preference for HCl, which is normally an aversive taste stimulus. A previous study reported a tendency for aged (28 months old) Sprague-Dawley rats to have less preference for citric acid than younger rats [14]. However, we found no significant age-related differences in the preference ratios for HCl, both vs. DW (Fig 2) and when two concentrations were presented simultaneously (Fig 4). The sour taste is thought to cause the action of protons on taste receptor cells, impacting taste transduction [27], though the precise mechanism is controversial. The HCl concentration used in our study may have been too strong of a stimulus to detect age-related differences.

A prior study using Fischer 344 rats demonstrated substantial chorda tympani nerve responses to taste stimuli even at 30 months of age [28]. The number of taste buds and taste bud diameter did not correlate with age in rats [29]. These reports support the idea that the altered taste preference in old-aged rats in the present study was not due to the function of the chorda tympani nerve. A study in 18-month-old mice, however, reported a significant reduction in taste bud size, the number of taste cells per bud, the number of taste cells expressing the sweet taste receptor, and the sweet taste-modulating hormone glucagon-like peptide-1 [16]. We have not yet examined whether the functions of other taste nerve components, such as the greater superficial petrosal nerve, superior laryngeal nerve, and glossopharyngeal nerve, are influenced by aging. Therefore, the results of the present study are not sufficient to rule out the possibility that aging induce the changes in the function of the peripheral taste system.

The present study evaluated taste preference using a 48-h two-bottle test method. The ingestion of taste substances affects subsequent consumption behaviors, referred to as the post-ingestive effects, via the gastrointestinal tract [30–32]. The perception of taste substances in the gastrointestinal tract causes changes in the level of feeding-related hormones, such as insulin and leptin [33]. Gastrointestinal motility [34–37] and levels of feeding-related hormones in the central nervous system [38] are altered with aging. These results might indicate that aging affects systemic physiological functions.

Taste and olfactory information are thought to converge in the central nervous system. Aging reportedly leads to alterations in the spontaneous function of the central nervous system [39, 40]. It is known that there are separable brain substrates underlying “wanting” (closely related to appetite) and “liking” (closely related to palatability) [41]. Another study reported that older rats exhibited decreased “wanting” and “liking” for a sweet reward in an incentive motivation task [42]. The central nervous system is heavily involved in the parameters of “wanting” and “liking” a sweet reward [41]. These data suggest that the age-related behavioral differences observed in the present study may be due to age-related changes in the central nervous system functions involved in ingestive behaviors. The future studies will identify the brain regions and neural circuits involved in the behavioral alterations observed among older rats.

## Conclusions

The present study revealed that the preference for sucrose and MSG decreases with age, whereas the preference for QHCl increases. We also found that old-aged rats consumed higher concentrations of sucrose, NaCl, MSG, and QHCl than younger rats, indicating that aging causes changes in taste preference. Although the aging-induced changes in taste preference are likely a result of alterations in the functions of peripheral and central organs, we found no age differences in the electrophysiological responses of the chorda tympani nerve, a peripheral taste nerve. This ruled out the possibility that the differences in taste preference among age groups are a result of altered function of the chorda tympani nerve. Therefore, to clarify the mechanisms underlying age-related changes in taste preference, future studies should investigate the effects of aging on the activities of other peripheral taste nerves, including the glossopharyngeal, greater superficial petrosal, and superior laryngeal nerves, and the function of the brain reward system involved in taste hedonics.

## Supporting information

S1 File The ARRIVE guidelines checklist. (PDF)

S2 File Raw data. (XLSX)
